# Management of subcutaneous abscesses: prospective cross-sectional study (MAGIC)

**DOI:** 10.1093/bjs/znae162

**Published:** 2024-08-20

**Authors:** White Rose Surgical Collaborative and MAGIC Collaborators, S J Chapman, J R Burke, J Helliwell, M Kowal, K Boag, M Ayres, L McMenamin, T Ho, J Carr-Wilkinson, A Peckham-Cooper, J Spackman, A Rashid, K Eng, V Boodhoo, T Williams, J Bundred, A Nessa, G Ramsay, G Greenlees, L Whittaker, N Pinsker, J Phillips, M Abu Sayed, A Kausar, J Spackman, R Ardley, A Rees, D Vimalachandran, L Henderson, D Watt, V Miu, R George, C English, B Stubbs, O Adepoju, L Nicol, L Rampersad, M S J Wilson, M Chauhan, S Shirol, M Abdelkarim, V Ochieng, A Golder, D Mansouri, D Liu, T Walker, M El Boghdady, K Adams, H Standing, C McNaught, W Ramadan, S Harris, A Ghoneima, A Saha, X Y Choo, M Alwahid, G Karagiannidis, J Pitt, G James, V Kanakala, B Down, G Bond-Smith, S Lamidi, A Raicu, A Hamadalnile, H Shanti, D Sapre, E Gemmill, W Y Chung, L B Samarakoon, S Ingram-Walpole, C Macutkiewicz, S Oo, R Harries, I El-Kharbotly, G Bashir, A Adesuyi, A Arain, N Abbud, N Shah, A Shalaby, B Appleton, B Dolaszynska, N Fazili, D Naumann, G Chillarge, N Smart, R Barnett, M Mulla, T Pelly, K Hancorn, J Honey, G Branagan, C Ezeme, R Newton, S Jones, A Peckham-Cooper, S Hodge, S Mehmood, M Bhardwaj, A Liaqat, S Wallace-King, W Carr, A Rahman, K Carswell, A S Opaluwa, S Hyder, Y Akula, S Robertson, C J Fairfield, F Maxwell, S Abdelrahman, S Abbott, H Fowler, N Pranesh, B Manku, A Baker, M Mehta, S Shirol, E Wates, R Fernandes R, M I K Malik, S Duff, N Lawrie, G Neelankavil Davis, D Dorkeh, E Moran, N Aruparayil, H Allott, J Creed, K Lehovsky, A Sallam, C Abdulrahman, S Puranik, P Suwichacherdchu, O M O'Connor, S Kaul, R Ghosh, B Davies, L Mamer, E McCarron, H Pendergast, J Corkerry, M Dowiedar, T Knowles, K Tin, L Demicoli, P Osei-Bonsu, J Balfour, A Varma, J Sikora, P Belaguthi, R Lee, L Berger, T Baron, S Myat, C Rowlands, O Oyekan, L Irabor Ezoba, S Tontus, T Panos, N Usmani, A Abdelhamid, S Kamil, A Maraqa, W Chantima, M Dyer, K Thane, S Yap, S Tompkins, A Asurlekar, P Polz, B Clark, D Low, K Robertson, O Hamid, Z L Hoo, Y W Wong, F Sheikh, V Satish, M Kronberga, S Suresan, M Kattakayam, A Ed Sabbagh, D Saleh, H Akhtar, S Gurung, S Bakare, E Ross, M Shahid, A Tweedie, C M Moen, M El-Farran, T Khaliq, B Peter, M Shams, J Fung, T Theivendrampillai, J Bundred, A Ang, R Ahmed, A Sherafat, K Annamalai, A Ali, V Kalogianni, A Hassan, W Aziz, B Srikumar, N Minhas, R Parks, S Hanif, A Farooq, M Salem, A Ali, G Davalos, A Hardy, Y Zhang, K Ngai, H Baker, J Ling-Soffe, M Mcmillan, D Thompson, A Dkhakhni, D Sapountzis, A Li, H Al-Siraj, S Khan, A Kuligowska, I Ogunmwonyi, R Broadbent, H George, A Rashid, I Zarad, M Osman, M Abdelsalam, A Gupta, S Moodalbyle, C Lewis, O Barrington M, Shams, C Jayasekara, K Lam, R Hwang, L Xu, A Ishak, W Protheroe, T Stylianou, E O Oladeji, H Anuff, E Husain, J Helliwell, J Holmes, N Shatwell, M Kishima M, F Issa, R Vashisht, G Bhardwaj, A Thaker, F Bandari, B Robertson-Jones, M Mostafa, T Nahiyan, N VinodKumar, B Altaf, A Sharma, O Khalil, A Mensah, E Ozekhome-Mike, F Ghafur, S Edensor, W Hodges, A Omran, W H Younas, Y Harris, E Spurring, F Osunlusi, C Okoronkwo, S Shah, E L Yan, R Hajdok, C Anderton, F Costigan, H Selvachandran, B David, A O'Sullivan, C Spreafico, M Phillips, S Arora, A Ahmed, M Ghotmi, R Mohamed, M Kawabata, A Gardner, I Atherley, A Chowdhury, J Kalliyath, K Manor, M Bamgboye, L Walker, J Law, E Reynolds, Z Bholah, R Castelhano

## Introduction

Subcutaneous abscesses are pus-filled cavities within the dermis and subdermal layers caused by a local bacterial infection, most commonly *Staphylococcus aureus*^1^. They are a common presentation, with an incidence of 433 per 100 000 presentations in community practice, leading to 236 per 100 000 admissions to hospital each year^2^. Surgical drainage is the conventional approach used to treat abscesses in the hospital setting. This aims to achieve complete evacuation of pus, thorough irrigation, and healing by secondary intention.

In the UK, no widely established guidance exists for the management of subcutaneous abscesses. The results of a survey of general surgeons in 2018 highlighted stark disparities in the everyday management of abscesses, such as in the choice of anaesthesia, the role of cavity packing, and the role of swabs for culture; in particular, uncertainty surrounding the use of general or local anaesthesia during surgical drainage was shown to exist^3^. A cohort study at a large teaching hospital later demonstrated the safety of a protocolized care pathway for drainage under local anaesthesia when applied to the majority (84%) of patients with uncomplicated abscesses^4^. The choice of anaesthesia remains of interest due to its potential impact on health resources, costs, the environment, and emergency care capacity.

Another uncertainty is the role of antibiotics after surgical drainage. In the previous survey, the majority of respondents indicated that antibiotics were only indicated sometimes (39.3%) or in rare circumstances (44.3%)^3^. This was in contrast to a recently published practice recommendation, advocating for the routine use of antimicrobial therapy after surgical drainage, specifically trimethoprim/sulfamethoxazole or clindamycin^5^. This recommendation was made owing to the results of a systematic review published in 2018, which demonstrated a modest reduction in abscess recurrence with antibiotics across 14 RCTs. In contrast, the review revealed an increase in the risk of gastrointestinal side effects, such as diarrhoea, and also raised concerns about other possible harms, such as from antimicrobial resistance^5,6^.

The aim of this study was to explore the management of subcutaneous abscesses in emergency surgical practice in the UK. In doing so, the study aimed to describe the UK experience of local anaesthesia as the primary anaesthetic modality for surgical drainage and the experience of antibiotic therapy as an adjuvant treatment after surgery.

## Methods

### Study governance

The study protocol was made prospectively available online. The Health Research Authority Decision Tool and a National Health Service (NHS) Research & Innovation Service confirmed that NHS Research Ethics Approval was not required. The study was registered with governance departments at each participating hospital, including confirmation of Caldecott Guardian approval for the safe transfer of non-identifiable data via the Research Electronic Data Capture (REDCap) platform^7^. The study is reported in line with the STROBE checklist^8^.

### Study design

A prospective, multicentre, observational study was delivered across NHS hospitals in the UK by the White Rose Surgical Collaborative. No change to routine clinical practice was made. The study opened for data inclusion on 1 September 2022 and closed on 1 December 2022, followed by a 60-day follow-up interval of routinely collected data. The inclusion interval was split into 1-month data collection intervals (*Table S1*), with participating hospital sites asked to contribute to at least one interval. A hospital-specific, 10-item survey exploring standards of management at each hospital was piloted and disseminated to local lead investigators.

### Study setting

Any secondary or tertiary care NHS hospital providing an acute emergency general surgery service in the UK was eligible to contribute. No limits on case volume or local policy for the management of acute subcutaneous abscesses were applied. Included patients were identified from any relevant acute care setting, including accident and emergency departments, acute surgical assessment and ambulatory units, and inpatient wards.

### Eligibility criteria

Any adult (greater than or equal to 18 years old) presenting with an acute subcutaneous abscess was eligible for inclusion. The abscess location had to be on the posterior neck, anterior or posterior thorax, abdomen, groin, buttock, natal cleft, or proximal limbs. All other abscesses, including peri-anal, breast, distal limb, and facial abscesses, were excluded, as these were considered to demand specialty-specific treatment not provided by an emergency general surgical practice. The remaining exclusion criteria comprised abscesses associated with a previous surgical site, abscesses with known body cavity communication, and lesions or ulcers considered to be of vascular aetiology.

### Outcome measures

The key outcomes of interest were abscess recurrence and return to hospital. Recurrence was defined as any recurrent abscess or surgical-site infection at the same anatomical site. Return to hospital was defined as any re-presentation, irrespective of whether this led to an inpatient admission. Both outcomes were measured using routinely collected data up to 60 days from the index treatment. For patients managed with antibiotics, the index treatment was the time of first antibiotics. For patients managed with surgery, the index treatment was the time of surgery.

### Explanatory variables

Explanatory variables were collected to provide a risk-adjusted estimate of outcomes. These included patient-related factors, such as age (years), sex, BMI (kg/m^2^), smoking status, and relevant co-morbidities (diabetes mellitus, hidradenitis suppurativa, and immunosuppressive therapeutics or conditions), and treatment-related factors, such as pre-hospital and hospital treatment (surgical drainage, needle aspiration, antibiotics, or no treatment). For patients undergoing surgery, anaesthetic and surgical variables were also collected, such as type of anaesthesia (general, local, or other), surgical setting (operating room, emergency department, or ward/treatment area), and operator grade.

### Statistical analysis

Patient and treatment characteristics for the entire cohort are presented descriptively as rates, mean(s.d.) values, or median (interquartile range (i.q.r.)) values. Effect estimates are presented as OR (95% c.i.) values. For the subgroup of patients undergoing surgical drainage, multivariable regression models were constructed for each of the clinical outcomes (abscess recurrence and return to hospital) to assess whether either of these was independently associated with: the use of postoperative antibiotics; the use of local anaesthetic; and packing of the abscess cavity. Explanatory variables were entered into the models using a stepwise approach. In addition, to examine whether variation existed between centres, multivariable models containing only patient-level variables along with corresponding multi-level models treating the admitting hospital as a random effect were generated for postoperative antibiotics, anaesthetic type, and abscess packing as outcomes. Residual and standard deviations of the random effects were examined and likelihood ratio tests were performed to identify differences in the models at the centre level. This generated *P* values to determine whether statistically significant centre-level variation existed for each outcome independent of case mix. Analyses were performed using SPSS^®^ (IBM, Armonk, NY, USA; version 22) and R statistical computing software (R Foundation for Statistical Computing, Vienna, Austria; version 4.3.3), with *P* < 0.050 indicative of statistical significance.

## Results

### Summary of recruitment

A total of 1637 records were collected from 54 NHS hospitals in the UK. After removal of duplicates, 1631 were considered in the analyses. The median number of records submitted per hospital was 27 (i.q.r. 15–44.5) during a median of 2 (i.q.r. 1–2) data collection intervals.

### Hospital-specific survey

A response to the survey was received from 54 of 54 hospitals (100%). A defined clinical pathway or protocol for the management of subcutaneous abscesses was in place at 14 hospitals (14 of 54; 25.9%). For surgery taking place in the operating room, dedicated abscess trays were used in 31 hospitals (31 of 54; 57.4%), of which 26 hospitals (26 of 31; 83.9%) used trays holding reusable instruments. For surgery taking place in an ambulatory unit, dedicated abscess trays were used in 11 hospitals (11 of 54; 20.4%), of which only 1 hospital (1 of 11; 9.1%) used trays holding reusable instruments.

### Patient characteristics

Of 1631 patients, a small majority were male (871 of 1631; 53.4%) and the most common age group was 26–35 years (351 of 1631; 21.5%). A total of 30.9% were smokers (490 of 1587), 15.6% were diabetic (254 of 1631), and 38.5% had a previous history of subcutaneous abscesses (628 of 1631). At presentation, the most common abscess positions were pilonidal (331 of 1631; 20.3%), posterior thorax (260 of 1631; 15.9%), and buttock (199 of 1631;12.2%), with a mean(s.d.) maximum abscess diameter of 4.2(2.3) cm. In half of cases, patients were treated with at least one course of antibiotics before presentation to hospital (867 of 1631; 53.2%) (*Table 1*).

**Table 1:** Patient characteristics

Patient characteristic	Category	Value
Age (years)	18–25	303 (18.6)
	26–35	351 (21.5)
	36–45	299 (18.3)
	46–55	285 (17.5)
	56–65	194 (11.9)
	>65	199 (12.2)
Sex	Male	871 (53.4)
	Female	760 (46.6)
Smoking status*	Current smoker	490 (30.9)
	Previous smoker	193 (12.2)
	Never smoked	904 (57.0)
BMI (kg/m^2^)*, mean(s.d.)	–	29.3(7.1)
Previous abscess	No	1002 (61.5)
	Yes	628 (38.5)
Hidradenitis suppurativa	No	1502 (92.1)
	Yes	129 (7.9)
Immunosuppression	No	1567 (96.1)
	Yes	64 (3.9)
Diabetes mellitus	No	1377 (84.4)
	Yes—diet controlled	38 (2.3)
	Yes—tablet controlled	139 (8.5)
	Yes—insulin controlled	77 (4.7)
Abscess position	Abdominal wall—anterior	136 (8.3)
	Abdominal wall—posterior	53 (3.2)
	Axilla	111 (6.8)
	Buttock	199 (12.2)
	Groin	190 (11.6)
	Lower limb—proximal	62 (3.8)
	Pilonidal	331 (20.3)
	Thorax—anterior	100 (6.1)
	Thorax—posterior	260 (15.9)
	Upper limb—proximal	118 (7.2)
	Other†	71 (4.4)
Maximum abscess diameter (cm)*, mean(s.d.)	–	4.2(2.3)
Timing of hospital presentation	Daytime (08.00–16.59)	1189 (72.9)
	Evening (17.00–21.59)	288 (17.7)
	Night (22.00–07.59)	154 (9.4)
Pre-hospital treatment	No pre-hospital treatment	717 (44.0)
	Antibiotics	867 (53.2)
	Surgery	29 (1.8)
	Other	18 (1.1)

Values are *n* (%) unless otherwise indicated. *Smoking status based on 1587 responses (*n* = 44 missing), BMI based on 1378 responses (*n* = 253 missing), and maximum abscess diameter based on 1458 responses (*n* = 146 missing). †Other comprises perineal abscesses (*n* = 7) and posterior neck abscesses (*n* = 64).

### Treatment characteristics

A total of 1351 patients underwent surgical drainage (1351 of 1631; 82.8%), with the remainder treated with needle aspiration (30 of 1631; 1.8%) or antibiotics alone (210 of 1631; 12.9%). Surgery was performed most commonly in the operating room (922 of 1351; 68.2%) or an ambulatory treatment area (409 of 1351; 30.0%). General anaesthesia was the most common anaesthetic modality (874 of 1351; 64.7%), followed by local anaesthesia (441 of 1351; 32.6%). The majority of abscess cavities were packed (1136 of 1351; 84.2%) and over half of the patients undergoing surgery received a short (less than 3 days) (123 of 1351; 9.1%) or longer (greater than or equal to 3 days) (579 of 1351; 42.9%) course of postoperative antibiotics (*Table 2*).

**Table 2:** Treatment characteristics

Treatment characteristic	Category	Value
Primary treatment	Surgical drainage	1351 (82.8)
	Needle aspiration	30 (1.8)
	Antibiotics only	210 (12.9)
	No treatment	31 (1.9)
	Other*	9 (0.6)
Timing of surgery†	First hospital presentation	917 (67.9)
	Planned return presentation	434 (32.1)
Preoperative antibiotics†	No	634 (47.0)
	Yes	716 (53.0)
Surgical setting†	Operating room	922 (68.2)
	Emergency department	20 (1.5)
	Ward/treatment area	409 (30.3)
Anaesthetic type†	General anaesthetic	874 (64.7)
	Local anaesthetic	441 (32.6)
	Other‡	36 (2.7)
Primary operator†	Consultant	75 (5.6)
	Junior surgical trainee	541 (40.1)
	Nurse practitioner	50 (3.7)
	Senior surgical trainee	682 (50.6)
Type of incision†	Cruciate	33 (2.5)
	Elliptical	238 (18.4)
	Linear	1024 (79.1)
Abscess packed†	No	213 (15.8)
	Yes	1136 (84.2)
Postoperative antibiotics†	No	648 (48.0)
	Yes—≥3 days total	579 (42.9)
	Yes—<3 days total	123 (9.1)

Values are *n* (%). Junior surgical trainee relates to postgraduate year 1–4 or junior clinical fellow and senior surgical trainee relates to postgraduate year 5–10 or senior clinical fellow. *Other comprises chlorhexidine/saline washout (*n* = 4), silver nitrate (*n* = 2), cold spray alone for symptomatic relief (*n* = 1), and surgery was planned, but patient declined or did not attend (*n* = 2). †Data relate to patients undergoing surgical drainage (*n* = 1351). ‡Other comprises spinal (*n* = 22), regional plexus block (*n* = 4), cold spray alone (*n* = 6); Entonox, and other inhalational gas alone (*n* = 4).

### Abscess recurrence

Recurrence occurred in 69 of 1351 patients (5.1%) undergoing surgical drainage and 17 of 280 patients (6.1%) undergoing any other treatment approach. For patients undergoing surgery, a higher BMI (OR 1.04, 95% c.i. 1.01 to 1.08; *P* = 0.015) and the presence of hidradenitis suppurativa (OR 2.64, 95% c.i. 1.13 to 5.62; *P*  *=* 0.016) were associated with a higher rate of recurrence within 60 days. Packing at the end of surgery (OR 0.38, 95% c.i. 0.21 to 0.72; *P* = 0.003) was associated with a lower rate of recurrence compared with not packing. Neither the use of local anaesthesia as the primary anaesthetic modality (OR 1.13, 95% c.i. 0.60 to 2.04; *P* = 0.698) or withholding postoperative antibiotics (OR 0.97, 95% c.i. 0.53 to 1.74; *P* = 0.906) was independently associated with a higher rate of recurrence (*Table 3*).

**Table 3:** Abscess recurrence within 60 days

Characteristic	Category	Univariable OR (95% c.i.), *P*	Multivariable OR (95% c.i.), *P*
Postoperative antibiotics	No	Reference	Reference
	Yes—≥3 days total	0.99 (0.56–1.76), 0.980	0.97 (0.53–1.74), 0.906
	Yes—<3 days total	0.62 (0.15–1.82, 0.446	0.64 (0.15–1.91), 0.478
Anaesthetic type	General anaesthetic	Reference	Reference
	Local anaesthetic	1.18 (0.65–2.08), 0.569	1.13 (0.60–2.04), 0.698
Age (years)	18–25	Reference	–
	26–35	1.32 (0.54–3.41), 0.544	–
	36–45	1.26 (0.48–3.37), 0.638	–
	46–55	1.41 (0.57–3.68), 0.464	–
	56–65	0.81 (0.24–2.48), 0.717	–
	>65	0.83 (0.25–2.54), 0.746	–
Sex	Male	Reference	–
	Female	0.84 (0.48–1.47), 0.553	–
Smoking status	Current smoker	Reference	Reference
	Previous smoker	0.37 (0.11–0.99), 0.073	0.38 (0.11–1.02), 0.080
	Never smoked	0.62 (0.35–1.11), 0.105	0.67 (0.37–1.23), 0.191
BMI (kg/m^2^), mean(s.d.)	–	1.05 (1.01–1.08), 0.006	1.04 (1.01–1.08), 0.015
Hidradenitis suppurativa	No	Reference	–
	Yes	3.02 (1.33–6.21), 0.004	2.64 (1.13–5.62), 0.016
Immunosuppression	No	Reference	–
	Yes	0.99 (0.16–3.35), 0.985	–
Diabetes mellitus	No	Reference	–
	Yes	1.19 (0.55–2.31), 0.638	–
Abscess position	Abdominal wall—anterior	Reference	–
	Abdominal wall—posterior	0.67 (0.03–4.73), 0.726	–
	Axilla	0.67 (0.09–3.54), 0.651	–
	Buttock	1.39 (0.41–5.45), 0.605	–
	Groin	2.11 (0.66–8.02), 0.225	–
	Lower limb—proximal	1.52 (0.20–8.16), 0.640	–
	Pilonidal	1.76 (0.61–6.34), 0.331	–
	Thorax—anterior	1.57 (0.36–6.85), 0.536	–
	Thorax—posterior	0.51 (0.12–2.22), 0.354	–
	Upper limb—proximal	1.68 (0.43–7.00), 0.452	–
	Other	0.51 (0.03–3.57), 0.553	–
Maximum abscess diameter (cm), mean(s.d)	–	0.98 (0.86–1.10), 0.776	–
Presence of cellulitis	No	Reference	–
	Yes	1.05 (0.56–1.88), 0.874	–
Timing of hospital presentation	Daytime (08.00–16.59)	Reference	–
	Evening (17.00–21.59)	0.81 (0.35–1.68), 0.605	–
	Night (22.00–07.59)	0.89 (0.30–2.11), 0.806	–
Timing of surgery	First hospital presentation	Reference	–
	Planned return presentation	0.99 (0.53–1.75), 0.961	–
Surgical setting	Operating room	Reference	–
	Emergency department	4.54 (0.67–18.5), 0.059	–
	Ward/treatment area	1.19 (0.64–2.14), 0.564	–
Primary operator	Consultant	Reference	–
	Junior surgical trainee	0.73 (0.27–2.56), 0.577	–
	Nurse practitioner	0.59 (0.08–3.16), 0.548)	–
	Senior surgical trainee	0.54 (0.20–1.90), 0.273	–
Abscess packed	No	Reference	–
	Yes	0.38 (0.21–0.72), 0.002	0.38 (0.20–0.74), 0.003

Analyses performed on cases without any missing data (*n* = 1047). Junior surgical trainee relates to postgraduate year 1–4 or junior clinical fellow and senior surgical trainee relates to postgraduate year 5–10 or senior clinical fellow.

### Return to hospital

Return to hospital within 60 days occurred in 215 of 1351 patients (15.9%) undergoing surgical drainage, including one patient with pneumonia (general anaesthetic), one patient with a deep collection (local anaesthetic), one patient with an acute coronary event (local anaesthetic), two patients with diabetic ketoacidosis (both general anaesthetic), and four patients with a haematoma (all general anaesthetic). Return to hospital occurred in 58 of 280 patients (20.7%) undergoing non-operative treatment approaches, including one due to a deep collection. The remainder of returns were due to other planned or non-emergency visits. For patients undergoing surgery, a higher BMI (OR 1.03, 95% c.i. 1.01 to 1.06; *P* = 0.003) and a prior history of abscesses (OR 1.45, 95% c.i. 1.04 to 2.01; *P*  *=* 0.029) were associated with more returns. Neither the use of local anaesthesia as the primary anaesthetic modality (OR 1.00, 95% c.i. 0.70 to 1.42; *P* = 0.995) or withholding postoperative antibiotics (OR 0.99, 95% c.i. 0.70 to 1.41; *P* = 0.976) was independently associated with a higher rate of return (*Table 4*).

**Table 4:** Return to hospital within 60 days

Characteristic	Category	Univariable OR (95% c.i.), *P*	Multivariable OR (95% c.i.), *P*
Postoperative antibiotics	No	Reference	Reference
	Yes—≥3 days total	1.11 (0.79–1.55), 0.547	0.99 (0.70–1.41), 0.976
	Yes—<3 days total	0.78 (0.39–1.44), 0.448	0.75 (0.37–1.41), 0.398
Anaesthetic type	General anaesthetic	Reference	Reference
	Local anaesthetic	0.97 (0.68–1.36), 0.841	1.00 (0.70–1.42), 0.995
Age (years)	18–25	Reference	–
	26–35	1.14 (0.69–1.91), 0.614	–
	36–45	0.84 (0.48–1.48), 0.551	–
	46–55	0.94 (0.55–1.63), 0.832	–
	56–65	1.07 (0.59–1.90), 0.830	–
	>65	0.79 (0.42–1.46), 0.454	–
Sex	Male	Reference	–
	Female	0.88 (0.64–1.22), 0.459	–
Smoking status	Current smoker	Reference	–
	Previous smoker	0.86 (0.50–1.43), 0.564	–
	Never smoked	0.82 (0.58–1.18), 0.278	–
BMI (kg/m^2^), mean(s.d.)	–	1.03 (1.01–1.06), 0.002	1.03 (1.01–1.06), 0.003
Hidradenitis suppurativa	No	Reference	–
	Yes	1.85 (1.04–3.16), 0.028	–
Immunosuppression	No	Reference	Reference
	Yes	1.92 (0.90–3.82), 0.073	1.95 (0.90–3.93), 0.073
Diabetes mellitus	No	Reference	–
	Yes	1.37 (0.90–2.04), 0.135	–
Abscess position	Abdominal wall—anterior	Reference	–
	Abdominal wall—posterior	0.53 (0.17–1.41), 0.232	–
	Axilla	0.47 (0.19–1.05), 0.075	–
	Buttock	0.51 (0.25–1.02), 0.059	–
	Groin	0.69 (0.35–1.36), 0.282	–
	Lower limb—proximal	1.04 (0.40–2.56), 0.928	–
	Upper limb—proximal	0.75 (0.35–1.56), 0.446	–
	Pilonidal	0.62 (0.34–1.13), 0.116	–
	Thorax—anterior	0.60 (0.26–1.34), 0.228	–
	Thorax—posterior	0.70 (0.38–1.28), 0.241	–
	Other	0.57 (0.21–1.39), 0.237	–
Maximum abscess diameter (cm), mean(s.d.)	–	1.02 (0.96–1.09), 0.477	–
Presence of cellulitis	No	Reference	Reference
	Yes	1.43 (1.01–2.00), 0.042	1.37 (0.96–1.94), 0.083
Timing of hospital presentation	Daytime (08.00–16.59)	Reference	–
	Evening (17.00–21.59)	0.77 (0.48–1.20), 0.265	–
	Night (22.00–07.59)	0.96 (0.54–1.61), 0.881	–
Timing of surgery	First hospital presentation	Reference	–
	Planned return presentation	0.90 (0.63–1.27), 0.560	–
Surgical setting	Operating room	Reference	–
	Emergency department	0.50 (0.03–2.63), 0.506	–
	Ward/treatment area	1.06 (0.74–1.50), 0.762	–
Primary operator	Consultant	Reference	–
	Junior surgical trainee	0.65 (0.33–1.35), 0.221	–
	Nurse practitioner	0.65 (0.22–1.79), 0.412	–
	Senior surgical trainee	0.68 (0.35–1.39), 0.261	–
Abscess packed	No	Reference	–
	Yes	0.80 (0.52–1.24), 0.304	–

Analyses performed on cases without any missing data (*n* = 1047). Junior surgical trainee relates to postgraduate year 1–4 or junior clinical fellow and senior surgical trainee relates to postgraduate year 5–10 or senior clinical fellow.

### Between-centre variation in practice

**Fig. 1:**
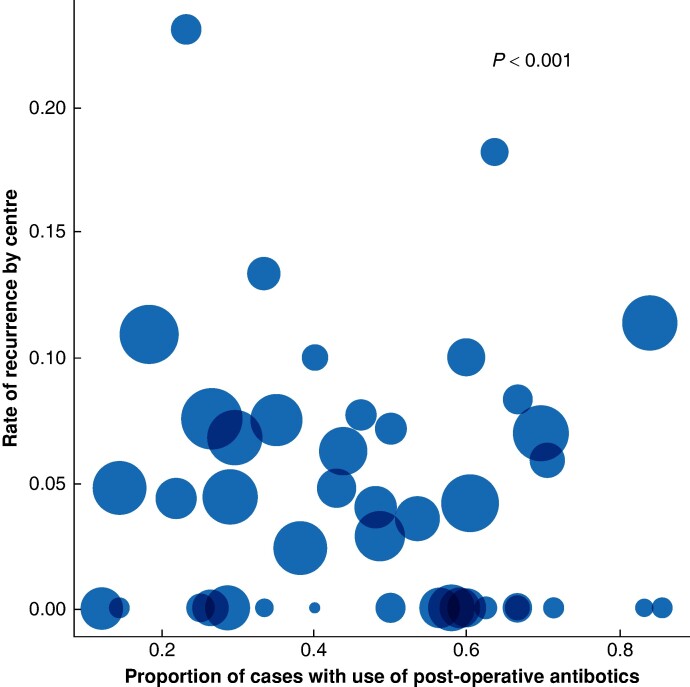
Variation in the use of postoperative antibiotics by centre The graph depicts the variation in the use of postoperative antibiotics *versus* the rate of recurrence. Circle size is proportionate to the number of participants included by each centre. The *P* value, generated using a likelihood ratio test, confirms statistically significant variation in the use of postoperative antibiotics at the centre level (see the Methods section).

**Fig. 2:**
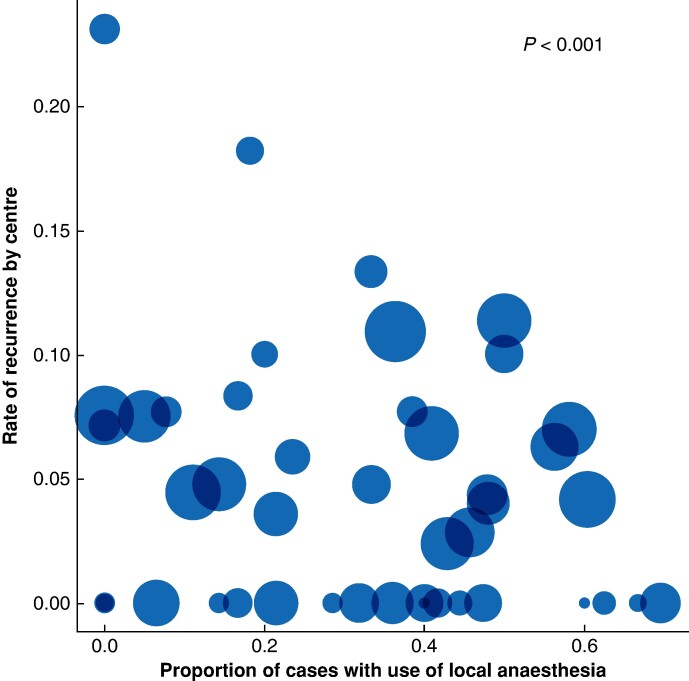
Variation in the use of local anaesthesia by centre The graph depicts the variation in the use of local anaesthesia *versus* the rate of recurrence. Circle size is proportionate to the number of participants included by each centre. The *P* value, generated using a likelihood ratio test, confirms statistically significant variation in the use of local anaesthesia at the centre level (see the Methods section).

**Fig. 3:**
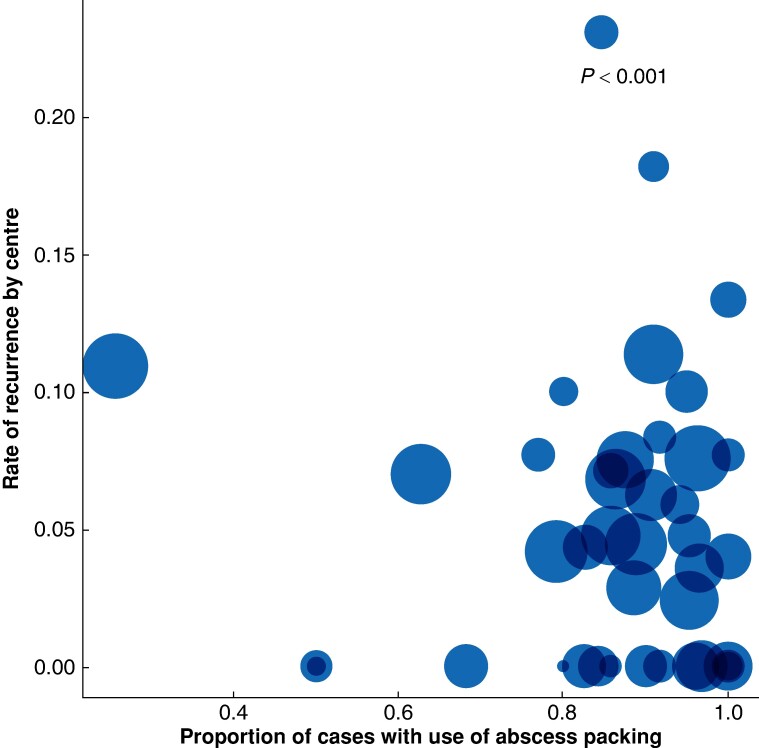
Variation in the use of abscess packing by centre The graph depicts the variation in the use of abscess packing *versus* the rate of recurrence. Circle size is proportionate to the number of participants included by each centre. The *P* value, generated using a likelihood ratio test, confirms statistically significant variation in the use of abscess packing at the centre level (see the Methods section).

There was wide variation between centres in the use of postoperative antibiotics (0–100%), local anaesthesia (0–69.6%), and abscess packing (0–100%), as shown in *Figs 1–3*. To explore this further, multivariable models (comprising only patient-level variables) and multi-level models (comprising patient-level and centre-level variables) were compared and are reported in full in *Table S2*. The multi-level models were significantly different from their respective multivariable models for anaesthetic type (*P* < 0.001), postoperative antibiotics (*P* < 0.001), and abscess packing (*P* < 0.001), suggesting a centre-level effect on each aspect of management. Overall, the results showed statistically significant variation in the choice of patient management strategies at the centre level, independent of the variation in case mix.

## Discussion

This study reveals wide variation in the management of acute subcutaneous abscesses in the UK. In particular, it reveals disparity in the choice of anaesthesia, the role of cavity packing, and the role of antibiotics. The data challenge the necessity of general anaesthesia as a default approach for surgical drainage, instead highlighting broad opportunities that exist in ambulatory or clinic-based settings with the use of local anaesthesia or other modalities that involve patients being awake. In the context of an observational study, the data show no clear indication of benefit of routine antimicrobial therapy after surgical drainage. The study provides a snapshot of practice specific to the UK, which should guide future research and other local quality improvement initiatives.

There is little evidence to guide the choice of anaesthesia during surgical drainage. A systematic review of six studies published between 2008 and 2014 showed that local and topical preparations appeared safe and effective, but only three of these focused on adult populations^9^. Despite a lack of robust evidence, local anaesthetic preparations are commonly used, as demonstrated by the present data. Indeed, one-third of all drainage procedures were undertaken using local anaesthesia alone and, in some hospitals, this was true for over half of their patients. Possible advantages of local anaesthesia in ambulatory settings include reduced admissions, fewer bed days, improved operating room flow, a quicker return to work and activities for patients, and reduced financial and environmental costs for health systems.

Whilst the use of antibiotics after surgical drainage is not routine in the UK, their use was advocated in a practice recommendation published in 2018^5^. This drew on a systematic review of 14 RCTs, demonstrating modest benefits with oral agents, such as trimethoprim/sulfamethoxazole or clindamycin. The data demonstrated a significant reduction in recurrence at 1 month (OR 0.48, 95% c.i. 0.30 to 0.77 (moderate quality)), as well as a reduction in re-hospitalization (OR 0.55, 95% c.i. 0.32 to 0.94 (moderate quality)). Conversely, the review showed that antibiotics increased gastrointestinal side effects (trimethoprim/sulfamethoxazole: OR 1.28, 95% c.i. 1.04 to 1.58 (moderate quality); and clindamycin: OR 2.29, 95% c.i. 1.35 to 3.88 (high quality)), including the incidence of diarrhoea^6^. In the present study, approximately half of all patients received antibiotics after surgery, with many receiving antibiotics for greater than or equal to 3 days. The modest benefits conferred by these antibiotics should be weighed carefully against potential harms of side effects and the societal impact of antimicrobial resistance^5^.

Abscess cavity packing is undertaken to prevent premature closure of the incision and, in turn, to reduce the risk of recurrence. The present data reveal a significant association between packing and reduced recurrence at 60 days. This contrasts with the results of other high-quality studies, such as the PPAC2 RCT, which demonstrated no difference in recurrence rates along with improved pain scores for patients whose perianal abscess cavity was not packed^10^. The results of the present study should be interpreted carefully, as no data are available on the duration of packing and whether this was done solely to achieve haemostasis after surgery. The data should, however, discourage extrapolation of previous results drawn from different populations and disease groups. The need for packing should remain a consideration after surgical drainage of subcutaneous non-perianal abscesses.

Limitations of the study are recognized. First, as an observational study of routinely collected data, it is likely that some events were missed, leading to under-reporting of key outcomes. This may have occurred if, for example, patients re-presented to community settings, alternative hospitals, or not at all^11^. Second, the study was unable to explore other outcomes that are probably important for patients and healthcare systems, such as pain, quality of life, cosmesis, and health economics. These remain an important target for future research. Third, owing to limitations of follow-up procedures, some return-to-hospital events may have been planned clinical reviews in hospital, particularly for the non-operative group; it was not possible to delineate these from emergency returns.

It is envisaged that the present data will initiate local discussions between relevant stakeholders around the management of subcutaneous abscesses. The role of local anaesthesia is a key consideration, particularly the opportunity it may present to expand ambulatory pathways and to reduce the environmental impact of the operating room. The role of antibiotics after surgical drainage is also a key consideration. The data should initiate local discussions between surgical and microbiology stakeholders with respect to the balance between treatment outcomes, side effects, and the risk of antimicrobial resistance. Local quality improvement initiatives are encouraged and the present data provide a baseline to guide such activities in the future.

## Collaborators

**White Rose Surgical Collaborative and MAGIC Collaborators**

S. J. Chapman, J.R. Burke, J. Helliwell, M. Kowal, K. Boag, M. Ayres, L. McMenamin, T. Ho, J. Carr-Wilkinson, A. Peckham-Cooper (Leeds Teaching Hospitals NHS Trust); J. Spackman (Chesterfield Royal Hospital NHS Foundation Trust); A. Rashid, K. Eng, V. Boodhoo (Sheffield Teaching Hospitals NHS Foundation Trust); T. Williams (Hull York Medical School); J. Bundred (Leeds Teaching Hospitals NHS Trust); A. Nessa, G. Ramsay (Aberdeen Royal Infirmary); G. Greenlees, L. Whittaker (Airedale General Hospital); N. Pinsker, J. Phillips (Barnsley Hospital); M. Abu Sayed, A. Kausar (Royal Blackburn Teaching Hospital); J. Spackman, R. Ardley (Chesterfield Royal Hospital NHS Foundation Trust); A. Rees, D. Vimalachandran (Countess of Chester Hospital); L. Henderson, D. Watt (Crosshouse Hospital); V. Miu, R. George (Doncaster Royal Infirmary); C. English, B. Stubbs (Dorset Country Hospital); O. Adepoju, L. Nicol (Dr Gray's Hospital); L. Rampersad, M.S.J. Wilson (Forth Valley Royal Hospital); M. Chauhan, S. Shirol (Frimley Park Hospital); M. Abdelkarim, V. Ochieng (Glan Clwyd Hospital); A. Golder, D. Mansouri (Glasgow Royal Infirmary); D. Liu, T. Walker (Gloucestershire Hospitals NHS Foundation Trust); M. El Boghdady, K. Adams (Guys and St. Thomas Hospital); H. Standing, C. McNaught (Harrogate District Hospital); W. Ramadan, S. Harris (The Hillingdon Hospitals NHS Foundation Trust); A. Ghoneima, A. Saha (Huddersfield Royal Infirmary); X. Y. Choo, M. Alwahid (Inverclyde Royal Hospital); G. Karagiannidis, J. Pitt (Ipswich Hospital NHS Trust); G. James, V. Kanakala (James Cook University Hospital); B. Down, G. Bond-Smith (John Radcliffe Hospital); S. Lamidi, A. Raicu (Kettering General Hospital); A. Hamadalnile, H. Shanti (Kings College Hospital); D. Sapre, E. Gemmill (Kings Mill Hospital); W. Y. Chung, L. B. Samarakoon (Leicester Royal Infirmary); S. Ingram-Walpole, C. Macutkiewicz (Manchester Royal Infirmary); S. Oo, R. Harries (Morriston Hospital); I. El-Kharbotly, G. Bashir (Newham University Hospital); A. Adesuyi, A. Arain (North Devon District Hospital); N. Abbud, N. Shah (Northern General Hospital); A. Shalaby, B. Appleton (Princess of Wales Hospital); B. Dolaszynska (Queen Elizabeth Hospital, Gateshead); N. Fazili, D. Naumann (Queen Elizabeth Hospital Birmingham); G. Chillarge, N. Smart (Royal Devon and Exeter Hospital); R. Barnett, M. Mulla (Royal Glamorgan Hospital); T. Pelly, K. Hancorn (Royal London Hospital); J. Honey, G. Branagan (Salisbury District General Hospital); C. Ezeme, R. Newton (St. Richard's Hospital); S. Jones, A. Peckham-Cooper (Leeds Teaching Hospitals NHS Trust); S. Hodge, S. Mehmood (Stepping Hill Hospital); M. Bhardwaj, A. Liaqat (Stoke Mandeville Hospital); S. Wallace-King, W. Carr (Sunderland Royal Hospital); A. Rahman, K. Carswell (Surrey and Sussex Healthcare NHS Trust); A. S. Opaluwa, S. Hyder (The Queen Elizabeth NHS Foundation Trust, Kings Lynn); Y. Akula, S. Robertson (University Hospitals Coventry and Warwickshire); C. J. Fairfield, F. Maxwell (University Hospital Wishaw); S. Abdelrahman, S. Abbott (Walsall Manor Hospital); H. Fowler, N. Pranesh (Warrington and Halton Teaching Hospitals NHS Foundation Trust); B. Manku, A. Baker (Warwick Hospital); M. Mehta, S. Shirol (Wexham Park Hospital); E. Wates, R. Fernandes R (William Harvey Hospital); M. I. K. Malik, S. Duff (Wythenshawe Hospital); N. Lawrie, G. Neelankavil Davis, D. Dorkeh (Aberdeen Royal Infirmary); E. Moran (Airedale General Hospital); N. Aruparayil, H. Allott, J. Creed, K. Lehovsky (Barnsley Hospital); A. Sallam, C. Abdulrahman, S. Puranik, P. Suwichacherdchu (Royal Blackburn Teaching Hospital); O. M. O'Connor, S. Kaul, R. Ghosh (Chesterfield Royal Hospital NHS Foundation Trust); B. Davies (Countess of Chester Hospital); L. Mamer, E. McCarron (Crosshouse Hospital); H. Pendergast, J. Corkerry, M. Dowiedar (Doncaster Royal Infirmary); T. Knowles, K. Tin, L. Demicoli (Dorset Country Hospital); P. Osei-Bonsu, J. Balfour, A. Varma (Dr Gray's Hospital); J. Sikora (Forth Valley Royal Hospital); P. Belaguthi, R. Lee, L. Berger, T. Baron, S. Myat, C. Rowlands, O. Oyekan, L. Irabor Ezoba, S. Tontus (Frimley Park Hospital); T. Panos, N. Usmani, A. Abdelhamid, S. Kamil, A. Maraqa, W. Chantima, M. Dyer, K. Thane, S, Yap (Glan Clwyd Hospital); S. Tompkins, A. Asurlekar, P. Polz, B. Clark, D. Low, K. Robertson (Glasgow Royal Infirmary); O. Hamid (Gloucestershire Hospitals NHS Foundation Trust) M. Pipe, N. Gadiyar (Guys and St. Thomas Hospital); Z. L. Hoo, Y. W. Wong (Harrogate District Hospital); F. Sheikh, V. Satish (The Hillingdon Hospitals NHS Foundation Trust); M. Kronberga, S. Suresan, M. Kattakayam, A. Ed Sabbagh, D. Saleh, H. Akhtar, S. Gurung, S. Bakare (Huddersfield Royal Infirmary); E. Ross, M. Shahid, A. Tweedie, C. M. Moen (Inverclyde Royal Hospital); M. El-Farran, T. Khaliq (Ipswich Hospital NHS Trust); B. Peter (James Cook University Hospital); M. Shams, J. Fung, T. Theivendrampillai,, J. Bundred, A. Ang, R. Ahmed (John Radcliffe Hospital); A. Sherafat, K. Annamalai, A. Ali (Kettering General Hospital); V. Kalogianni, A. Hassan (Kings College Hospital); W. Aziz, B. Srikumar, N. Minhas, R. Parks, S. Hanif, A. Farooq, M. Salem, A. Ali (Kings Mill Hospital); G. Davalos (Leicester Royal Infirmary); A. Hardy, Y. Zhang (Manchester Royal Infirmary); K. Ngai, H. Baker, J. Ling-Soffe, M. Mcmillan, D. Thompson (Morriston Hospital); A. Dkhakhni, D. Sapountzis (Newham University Hospital); A. Li, H. Al-Siraj (North Devon District Hospital); S. Khan, A. Kuligowska, I. Ogunmwonyi, R. Broadbent, H. George, A. Rashid (Northern General Hospital); I. Zarad, M. Osman, M. Abdelsalam (Princess of Wales Hospital); A. Gupta (Queen Elizabeth Hospital, Gateshead); S. Moodalbyle, C. Lewis (Queen Elizabeth Hospital Birmingham); O. Barrington M, Shams (Royal Devon and Exeter Hospital); C. Jayasekara, K. Lam (Royal Glamorgan Hospital); R. Hwang, L. Xu, A. Ishak (Royal London Hospital); W. Protheroe, T. Stylianou (Salisbury District General Hospital); E. O. Oladeji, H. Anuff, E. Husain (St. Richard's Hospital); J. Helliwell, J. Holmes, N. Shatwell (Leeds Teaching Hospitals NHS Trust); M. Kishima M (Stepping Hill Hospital); F. Issa, R. Vashisht, G. Bhardwaj (Stoke Mandeville Hospital); A. Thaker, F. Bandari (Sunderland Royal Hospital); B. Robertson-Jones, M. Mostafa, T. Nahiyan (Surrey and Sussex Healthcare NHS Trust); N. VinodKumar, B. Altaf, A. Sharma, O. Khalil, A. Mensah, E. Ozekhome-Mike (The Queen Elizabeth NHS Foundation Trust, Kings Lynn); F. Ghafur, S. Edensor, W. Hodges (University Hospitals Coventry and Warwickshire); A. Omran, W. H. Younas, Y. Harris (University Hospital Wishaw); E. Spurring, F. Osunlusi, C. Okoronkwo, S. Shah, E. L. Yan, R. Hajdok (Walsall Manor Hospital); C. Anderton, F. Costigan, H. Selvachandran, B. David (Warrington and Halton Teaching Hospitals NHS Foundation Trust); A. O'Sullivan, C. Spreafico, M. Phillips (Warwick Hospital); S. Arora, A. Ahmed, M. Ghotmi, R. Mohamed (Wexham Park Hospital); M. Kawabata, A. Gardner, I. Atherley, A. Chowdhury, J. Kalliyath (William Harvey Hospital); K. Manor, M. Bamgboye, L. Walker, J. Law, E. Reynolds, Z. Bholah, R. Castelhano (Wythenshawe Hospital).

## Supplementary Material

znae162_Supplementary_Data

## Data Availability

Requests for data sharing will be considered by the study management group upon receipt of a statistical analysis plan.
