# Supplementary material for: Management of subcutaneous abscesses: prospective cross-sectional study (MAGIC)
Source: Br J Surg. 2024 Aug 20;111(8):znae162. doi: 10.1093/bjs/znae162 (PMC11334058; doi:10.1093/bjs/znae162)
Supplement: znae162_Supplementary_Data [file znae162_supplementary_data.docx]

**Management of subcutaneous abscesses: Prospective cross-sectional study (MAGIC)**

White Rose Surgical Collaborative and MAGIC Collaborators*
**Collaborating members are shown at the end of the manuscript.*

**Correspondence on behalf of the White Rose Surgical Collaborative:**

Stephen J Chapman, Room 7.16 Clinical Sciences Building, St. James’s University Hospital, University of Leeds, LS2 9JT, United Kingdom

Correspondence: [s.chapman@leeds.ac.uk](mailto:s.chapman@leeds.ac.uk) @SJ_Chapman

**Supplementary Materials - Index**

| **Supplementary Methods** |  |
| --- | --- |
| Assessment of between-unit variation | *pag. 3* |
| **Supplementary Results** |  |
| Choice of anaesthetic type | *pag. 4* |
| Post-operative antibiotics prescribing  Abscess packing | *pag. 4*  *pag. 4* |
|  |  |
| **Supplementary Figures and Tables** |  |
| Table S1 – Data collection periods | *pag. 5* |
| Table S2 – Models exploring centre-level variation | *pag. 6* |
|  |  |
| **Supplementary Appendix**  Appendix S1 – Collaborative Authorship | *pag. 7* |

**Supplementary Methods**

*Assessment of between-unit variation*

To assess variation in abscess management between centres, patient-level factors associated with post-operative antibiotic prescribing, use of local anaesthetic, and abscess packing were identified using binary logistic regression models. All patient-level factors were considered for entry into multivariable models predicting antibiotic prescribing, anaesthetic type or abscess packing. Variables were entered into multivariable models using a stepwise approach to variable selection. After generation of final multivariable models, multilevel models, including the treating centre as a random effect and the original multivariable model of patient-level factors were generated. Residual and standard deviations of the random effects were examined and likelihood ratio tests were performed to identify differences in the models at the centre level.

**Supplementary Results**

*Choice of anaesthetic type*

Patient sex, diagnosis of hidradenitis, immunosuppression, abscess position and diameter, timing of presentation, and primary operator were associated with choice of anaesthetic type. On likelihood ratio testing, multilevel modelling including the treating hospital as the random effect in addition to patient-level variables, was significantly different to the multivariable model p<0.001 suggesting an effect of the centre on anaesthetic choice independent of case mix (Table S2)

*Post-operative antibiotic prescribing*

Patient sex, smoking status, presence of diabetes, abscess diameter, presence of cellulitis, antibiotics prior to surgery, previous surgical management of abscess, and timing of surgery were associated with the use of antibiotics after surgery. On likelihood ratio testing, multilevel modelling including the treating hospital as the random effect in addition to patient-level variables, was significantly different to the multivariable model p<0.001 suggesting an effect of the centre on antibiotic prescribing independent of case mix (Table S2)

*Abscess Packing*

previous surgical management of abscess, hidradenitis suppurative, abscess position, presence of cellulitis, setting of operation, and primary operator were associated with choice of whether to pack the abscess. On likelihood ratio testing, multilevel modelling including the treating hospital as the random effect in addition to patient-level variables, was significantly different to the multivariable model p<0.001 suggesting an effect of the centre on abscess packing independent of case mix (Table S2)

**Table S1 – Data Collection Periods**

| **Time Period** |  |
| --- | --- |
| Data Collection Period 1 | 08:00AM 1^st^ Sept 2022 – 07:59AM 1^st^ Oct 2022 |
| Data Collection Period 2 | 08:00AM 1^st^ Oct 2022 – 07:59AM 1^st^ Nov 2022 |
| Data Collection Period 3 | 08:00AM 1^st^ Nov 2022 – 07:59AM 1^st^ Dec 2022 |

|  |  | Anaesthetic Type (GA vs local) | | Post-op Antibiotics (Yes vs No) | | Abscess Packed (Yes vs No) | |
| --- | --- | --- | --- | --- | --- | --- | --- |
|  |  | ***OR(Multivariable)*** | ***OR (multilevel)*** | ***OR(Multivariable)*** | ***OR (multilevel)*** | ***OR(Multivariable)*** | ***OR (multilevel)*** |
| Anaesthetic type | *GA* | - | - | - | - | - | - |
|  | *Local* | - | - | 2.78 (1.99-3.91, p<0.001) | 2.68 (1.84-3.92, p<0.001) | - | - |
| Sex | *Male* | - | - | - | - | - | - |
|  | *Female* | 0.66 (0.48-0.91, p=0.011) | 0.56 (0.39-0.82, p=0.003) | - | - | - | - |
| Smoking | *Current Smoker* | - | - | - | - | - | - |
|  | *Previous Smoker* | - | - | 1.49 (0.95-2.35, p=0.086) | 1.62 (0.99-2.65, p=0.053) | - | - |
|  | *Never Smoked* | - | - | 1.40 (1.02-1.92, p=0.036) | 1.51 (1.07-2.12, p=0.018) | - | - |
| Previous abscess surgery | *No* | - | - | - | - | - | - |
|  | *Yes* | - | - | 6.28 (1.31-46.06, p=0.034) | 6.86 (1.06-44.28, p=0.043) | 0.60 (0.41-0.89, p=0.011) | 0.53 (0.35-0.83, p=0.005) |
| Hidradenitis | *No* | - | - | - | - | - | - |
|  | *Yes* | 0.56 (0.28-1.05, p=0.080) | 0.52 (0.25-1.08, p=0.081) | - | - | 1.98 (0.87-5.03, p=0.125) | 2.14 (0.79-5.74, p=0.132) |
| Immunosuppression | *No* | - | - | - | - | - | - |
|  | *Yes* | 2.03 (0.93-4.42, p=0.074) | 3.09 (1.17-8.21, p=0.023) | - | - | - | - |
| Diabetes | *No* | - | - | - | - | - | - |
|  | *Yes* | - | - | 2.04 (1.38-3.06, p<0.001) | 2.12 (1.38-3.27, p=0.001) | - | - |
| Abscess Position | *Abdominal Wall - Anterior* | - | - | - | - | - | - |
|  | *Abdominal Wall - Posterior* | 0.76 (0.32-1.80, p=0.543) | 0.72 (0.27-1.93, p=0.518) | - | - | 1.72 (0.47-8.30, p=0.449) | 1.83 (0.42-7.92, p=0.421) |
|  | *Axilla* | 0.49 (0.23-1.01, p=0.056) | 0.38 (0.17-0.87, p=0.023) | - | - | 2.08 (0.64-8.09, p=0.244) | 2.15 (0.56-8.29, p=0.266) |
|  | *Buttock* | 0.22 (0.11-0.44, p<0.001) | 0.14 (0.06-0.31, p<0.001) | - | - | 0.97 (0.39-2.37, p=0.955) | 1.21 (0.46-3.17, p=0.699) |
|  | *Groin* | 0.36 (0.18-0.70, p=0.003) | 0.29 (0.14-0.60, p=0.001) | - | - | 0.47 (0.20-1.08, p=0.079) | 0.60 (0.24-1.50, p=0.276) |
|  | *Lower Limb - Proximal* | 0.43 (0.16-1.06, p=0.075) | 0.30 (0.11-0.87, p=0.026) | - | - | 1.52 (0.41-7.34, p=0.559) | 1.76 (0.38-8.15, p=0.468) |
|  | *Pilonidal* | 0.11 (0.06-0.22, p<0.001) | 0.07 (0.03-0.15, p<0.001) | - | - | 1.00 (0.43-2.23, p=0.997) | 1.41 (0.58-3.44, p=0.445) |
|  | *Thorax - Anterior* | 1.63 (0.81-3.31, p=0.175) | 1.81 (0.79-4.13, p=0.159) | - | - | 0.30 (0.12-0.71, p=0.007) | 0.37 (0.14-0.96, p=0.042) |
|  | *Thorax - Posterior* | 1.27 (0.73-2.21, p=0.401) | 1.38 (0.73-2.61, p=0.324) | - | - | 0.86 (0.38-1.84, p=0.714) | 1.18 (0.51-2.75, p=0.700) |
|  | *Upper Limb - Proximal* | 0.78 (0.38-1.56, p=0.477) | 0.71 (0.32-1.57, p=0.398) | - | - | 0.42 (0.17-1.01, p=0.054) | 0.59 (0.22-1.58, p=0.292) |
| Abscess Diameter | *Mean (SD)* | 0.76 (0.70-0.82, p<0.001) | 0.72 (0.65-0.79, p<0.001) | 1.09 (1.02-1.16, p=0.009) | 1.12 (1.05-1.21, p=0.001) | - | - |
| Cellulitis | *No* | - | - | - | - | - | - |
|  | *Yes* | - | - | 2.34 (1.73-3.20, p<0.001) | 2.22 (1.58-3.13, p<0.001) | 1.58 (1.02-2.50, p=0.043) | 1.39 (0.85-2.28, p=0.194) |
| Timing of Presentation | *Daytime (0800-1659)* | - | - | - | - | - | - |
|  | *Out of hours/evening (1700-2159)* | 0.70 (0.46-1.07, p=0.108) | 0.70 (0.44-1.12, p=0.141) | - | - | - | - |
|  | *Out of hours/night (2200-0759)* | 0.55 (0.31-0.94, p=0.033) | 0.59 (0.31-1.10, p=0.095) | - | - | - | - |
| Antibiotics prior to presentation | *No* | - | - | - | - | - | - |
|  | *Yes* | - | - | 0.76 (0.58-1.00, p=0.055) | 0.83 (0.61-1.13, p=0.231) | - | - |
| Antibiotics between presentation and surgery | *No* | - | - | - | - | - | - |
|  | *Yes* | - | - | 3.81 (2.83-5.17, p<0.001) | 4.16 (2.97-5.84, p<0.001) | - | - |
| Timing of Surgery | *Surgery performed at first hospital presentation* | - | - | - | - | - | - |
|  | *Surgery performed at planned return presentation* | - | - | 0.59 (0.43-0.80, p=0.001) | 0.57 (0.40-0.81, p=0.002) | - | - |
| Surgical Setting | *Operating Room* | - | - | - | - | - | - |
|  | *Emergency Department* | - | - | - | - | 0.24 (0.06-1.11, p=0.054) | 0.17 (0.03-0.80, p=0.025) |
|  | *Ward/Treatment Area* | - | - | - | - | 0.56 (0.36-0.86, p=0.008) | 0.47 (0.28-0.79, p=0.004) |
| Primary operator | *Consultant* | - | - | - | - | - | - |
|  | *Junior Surgical Trainee (FY1-CT2, Clinical Fellow)* | 0.41 (0.21-0.78, p=0.007) | 0.47 (0.21-1.03, p=0.058) | - | - | 0.39 (0.14-0.92, p=0.047) | 0.68 (0.24-1.95, p=0.478) |
|  | *Nurse practitioner* | 26.19 (4.58-502.93, p=0.003) | 27.68 (2.84-270.28, p=0.004) | - | - | 0.43 (0.13-1.30, p=0.145) | 0.70 (0.19-2.60, p=0.590) |
|  | *Senior Surgical Trainee (ST3-8, Senior Fellow)* | 0.22 (0.11-0.41, p<0.001) | 0.27 (0.12-0.59, p=0.001) | - | - | 0.77 (0.27-1.84, p=0.577) | 1.00 (0.35-2.86, p=1.000) |

**Table S2 – Models exploring centre-level variation**

*Multivariable models were developed to predict anaesthetic type, the use of post-operative antibiotics, and abscess packing, according to the Methods. Patient-level factors were run with and without a centre level variable (multivariable and multilevel models). P-values from likelihood ratio tests comparing models with and without centre level variables were: Anaesthetic type p<0.001, Post-operative antibiotics p<0.001, abscess packing p<0.001*

**Appendix S1 – Collaborative Authorship**

**Study Steering Committee:** Chapman SJ*, Burke JR,* Helliwell J, Kowal M, Boag K, Ayres M, McMenamin L, Ho T, Carr-Wilkinson J, Peckham-Cooper A (Leeds Teaching Hospitals NHS Trust), Spackman J (Chesterfield Royal Hospital NHS Foundation Trust), Rashid A, Eng K, Boodhoo V (Sheffield Teaching Hospitals NHS Foundation Trust), Williams T (Hull York Medical School).

**Statistical Guarantor**: Bundred J (Leeds Teaching Hospitals NHS Trust)

**Local Leads:** Nessa A, Ramsay G (Aberdeen Royal Infirmary), Greenlees G, Whittaker L (Airedale General Hospital), Pinsker N, Phillips J (Barnsley Hospital), Abu Sayed M, Kausar A (Royal Blackburn Teaching Hospital), Spackman J, Ardley R (Chesterfield Royal Hospital NHS Foundation Trust), Rees A, Vimalachandran D (Countess of Chester Hospital), Henderson L, Watt D (Crosshouse Hospital), Miu V, George R (Doncaster Royal Infirmary), English C, Stubbs B (Dorset Country Hospital), Adepoju O, Nicol L (Dr Gray’s Hospital), Rampersad L, Wilson MSJ (Forth Valley Royal Hospital), Chauhan M, Shirol S (Frimley Park Hospital), Abdelkarim M, Ochieng V (Glan Clwyd Hospital), Golder A, Mansouri D (Glasgow Royal Infirmary), Liu D, Walker T (Gloucestershire Hospitals NHS Foundation Trust), El Boghdady M, Adams K (Guys and St. Thomas Hospital), Standing H, McNaught C (Harrogate District Hospital), Ramadan W, Harris S (The Hillingdon Hospitals NHS Foundation Trust), Ghoneima A, Saha A (Huddersfield Royal Infirmary), Choo XY, Alwahid M (Inverclyde Royal Hospital), Karagiannidis G, Pitt J (Ipswich Hospital NHS Trust), James G, Kanakala V (James Cook University Hospital), Down B, Bond-Smith G (John Radcliffe Hospital), Lamidi S, Raicu A (Kettering General Hospital), Hamadalnile A, Shanti H (Kings College Hospital), Sapre D, Gemmill E (Kings Mill Hospital), Chung WY, Samarakoon LB (Leicester Royal Infirmary), Ingram-Walpole S, Macutkiewicz C (Manchester Royal Infirmary), Oo S, Harries R (Morriston Hospital), El-Kharbotly I, Bashir G (Newham University Hospital), Adesuyi A, Arain A (North Devon District Hospital), Abbud N, Shah N (Northern General Hospital), Shalaby A, Appleton B (Princess of Wales Hospital), Dolaszynska B (Queen Elizabeth Hospital, Gateshead), Fazili N, Naumann D (Queen Elizabeth Hospital Birmingham), Chillarge G, Smart N (Royal Devon and Exeter Hospital), Barnett R, Mulla M (Royal Glamorgan Hospital), Pelly T, Hancorn K (Royal London Hospital), Honey J, Branagan G (Salisbury District General Hospital), Ezeme C, Newton R (St. Richard’s Hospital), Jones S, Peckham-Cooper A (Leeds Teaching Hospitals NHS Trust), Hodge S, Mehmood S (Stepping Hill Hospital), Bhardwaj M, Liaqat A (Stoke Mandeville Hospital), Wallace-King S, Carr W (Sunderland Royal Hospital), Rahman A, Carswell K (Surrey and Sussex Healthcare NHS Trust), Opaluwa AS, Hyder S (The Queen Elizabeth NHS Foundation Trust, Kings Lynn), Akula Y, Robertson S (University Hospitals Coventry and Warwickshire), Fairfield CJ, Maxwell F (University Hospital Wishaw), Abdelrahman S, Abbott S (Walsall Manor Hospital), Fowler H, Pranesh N (Warrington and Halton Teaching Hospitals NHS Foundation Trust), Manku B, Baker A (Warwick Hospital), Mehta M, Shirol S (Wexham Park Hospital), Wates E, Fernandes R (William Harvey Hospital), Malik MIK, Duff S (Wythenshawe Hospital)

**Local Collaborators:** Lawrie N, Neelankavil Davis G, Dorkeh D (Aberdeen Royal Infirmary), Moran E (Airedale General Hospital), Aruparayil N, Allott H, Creed J, Lehovsky K (Barnsley Hospital), Sallam A, Abdulrahman C, Puranik S, Suwichacherdchu P (Royal Blackburn Teaching Hospital), O’Connor OM, Kaul S, Ghosh R (Chesterfield Royal Hospital NHS Foundation Trust), Davies B (Countess of Chester Hospital), Mamer L, McCarron E (Crosshouse Hospital), Pendergast H, Corkerry J, Dowiedar M (Doncaster Royal Infirmary), Knowles T, Tin K, Demicoli L (Dorset Country Hospital), Osei-Bonsu P, Balfour J, Varma A (Dr Gray’s Hospital), Sikora J (Forth Valley Royal Hospital), Belaguthi P, Lee R, Berger L, Baron T, Myat S, Rowlands C, Oyekan O, Irabor Ezoba L, Tontus S (Frimley Park Hospital), Panos T, Usmani N, Abdelhamid A, Kamil S, Maraqa A, Chantima W, Dyer M, Thane K, Yap S (Glan Clwyd Hospital), Tompkins S, Asurlekar A, Polz P, Clark B, Low D, Robertson K (Glasgow Royal Infirmary), Hamid O (Gloucestershire Hospitals NHS Foundation Trust) Pipe M, Gadiyar N (Guys and St. Thomas Hospital), Hoo ZL, Wong YW (Harrogate District Hospital), Sheikh F, Satish V (The Hillingdon Hospitals NHS Foundation Trust), Kronberga M, Suresan S, Kattakayam M, Ed Sabbagh A, Saleh D, Akhtar H, Gurung S, Bakare S (Huddersfield Royal Infirmary), Ross E, Shahid M, Tweedie A, Moen CM (Inverclyde Royal Hospital), El-Farran M, Khaliq T (Ipswich Hospital NHS Trust), Peter B (James Cook University Hospital), Shams M, Fung J, Theivendrampillai, T, Bundred J, Ang A, Ahmed R (John Radcliffe Hospital), Sherafat A, Annamalai K, Ali A (Kettering General Hospital), Kalogianni V, Hassan A (Kings College Hospital), Aziz W, Srikumar B, Minhas N, Parks R, Hanif S, Farooq A, Salem M, Ali A (Kings Mill Hospital), Davalos G (Leicester Royal Infirmary), Hardy A, Zhang Y (Manchester Royal Infirmary), Ngai K, Baker H, Ling-Soffe J, Mcmillan M, Thompson D (Morriston Hospital), Dkhakhni A, Sapountzis D (Newham University Hospital), Li A, Al-Siraj H (North Devon District Hospital), Khan S, Kuligowska A, Ogunmwonyi I, Broadbent R, George H, Rashid A (Northern General Hospital), Zarad I, Osman M, Abdelsalam M (Princess of Wales Hospital), Gupta A (Queen Elizabeth Hospital, Gateshead), Moodalbyle S, Lewis C (Queen Elizabeth Hospital Birmingham), Barrington M, Shams O (Royal Devon and Exeter Hospital), Jayasekara C, Lam K (Royal Glamorgan Hospital), Hwang R, Xu L, Ishak A (Royal London Hospital), Protheroe W, Stylianou T (Salisbury District General Hospital), Oladeji EO, Anuff H, Husain E (St. Richard’s Hospital), Helliwell J, Holmes J, Shatwell N (Leeds Teaching Hospitals NHS Trust), Kishima M (Stepping Hill Hospital), Issa F, Vashisht R, Bhardwaj G (Stoke Mandeville Hospital), Thaker A, Bandari F (Sunderland Royal Hospital), Robertson-Jones B, Mostafa M, Nahiyan T (Surrey and Sussex Healthcare NHS Trust), VinodKumar N, Altaf B, Sharma A, Khalil O, Mensah A, Ozekhome-Mike, E (The Queen Elizabeth NHS Foundation Trust, Kings Lynn), Ghafur F, Edensor S, Hodges W (University Hospitals Coventry and Warwickshire), Omran A, Younas WH, Harris Y (University Hospital Wishaw), Spurring E, Osunlusi F, Okoronkwo C, Shah S, Yan EL, Hajdok R (Walsall Manor Hospital), Anderton C, Costigan F, Selvachandran H, David B (Warrington and Halton Teaching Hospitals NHS Foundation Trust), O’Sullivan A, Spreafico C, Phillips M (Warwick Hospital), Arora S, Ahmed A, Ghotmi M, Mohamed R (Wexham Park Hospital), Kawabata M, Gardner A, Atherley I, Chowdhury A, Kalliyath J (William Harvey Hospital), Manor K, Bamgboye M, Walker L, Law J, Reynolds E, Bholah Z, Castelhano R (Wythenshawe Hospital)
